# Polyesters Based on Linoleic Acid for Biolubricant Basestocks: Low-Temperature, Tribological and Rheological Properties

**DOI:** 10.1371/journal.pone.0151603

**Published:** 2016-03-23

**Authors:** Bashar Mudhaffar Abdullah, Saiful Irwan Zubairi, Hasniza Zaman Huri, Nany Hairunisa, Emad Yousif, Roma Choudhury Basu

**Affiliations:** 1 Clinical Investigation Centre, University Malaya Medical Centre, 13th Floor Main Tower, Lembah Pantai, Kuala Lumpur, Malaysia; 2 School of Chemical Sciences and Food Technology, Faculty of Science and Technology, Universiti Kebangsaan Malaysia, UKM Bangi, Selangor, Malaysia; 3 Department of Pharmacy, Faculty of Medicine, University of Malaya, Kuala Lumpur, Malaysia; 4 Department of Chemistry, College of Science, Al-Nahrain University, Baghdad, Iraq; USDA-ARS, UNITED STATES

## Abstract

Presently, plant oils which contain high percentage of linoleic acid **1** are perceived to be a viable alternative to mineral oil for biolubricant applications due to their biodegradability and technical properties. In order to get biodegradable lubricant, triester derivatives compounds (**1**–**5**) were synthesized and characterized. The processes involved were monoepoxidation of linoleic acid **2**, oxirane ring opening **3**, esterification **4** and acylation **5**. The structures of the products were confirmed by FTIR, ^1^H and ^13^C-NMR and LC-MS. The results that showed lowest temperature properties were obtained for triester **5**, with a pour point value (PP) of -73°C, highest onset temperature (260°C) and lowest volatility at 0.30%. Viscosity index (VI) increased for the ester’s synthetic compounds (**2**, **3**, **4**, **5**), while the PP decreased. This behavior is the result of the increase of the chain length of the branching agents. Triester based linoleic acid has improved properties such as low-temperature and tribological properties. These results will make it feasible for plant oil to be used for biolubricants, fuels in chain saws, transmission oil and brake fluid.

## Introduction

In this new age of techno-shift, the growing concern is the effort to protect the environment from the hazards of petroleum-based products. The poor biodegradability of petroleum oil pressurizes the industry to develop eco-friendly biodegradable lubricants based plant oils [1]. Even though plant oils possess excellent biodegradability, renewability and lubricating performance in industrial applications; there are two major issues of concern: poor low-temperature and oxidative stability properties for utilization as biolubricant base stocks [2, 3]. To overcome these problems, the chemical and biological modification of plant oils is the most feasible choice [4]. Many researchers and scientists have began to consider different types of natural material such as plant oils, animal fats, and organic waste oil as the starting materials for the synthesis of biolubricants for applications in food-industry, agricultural equipment, metal working fluids, biodegradable grease and others [5].

Fundamentally oleochemical products may be classified as fatty acids, fatty alcohols, and glycerine, which are mostly obtained directly from plant oils with linear or branch carbon chains [4]. Transforming unsaturated fatty acids such as linoleic acid (LA) to other groups could improve the oxidative stability, while attaching the alkyl side chains might improve low-temperature performance [6].

The epoxidation reaction is the forming of an oxirane ring by the oxidation of double bonds of unsaturated fatty acids [7] and could be varied due to the possibility of mono- or di-epoxides depending on the type of unsaturated fatty acids [8]. Carbon double bonds of unsaturated fatty acids can be epoxidized by different methods according to the feedstock, oxidation reagent, catalysts (acid catalysis or lipase catalysis) and solvents [7]. Epoxides are among the most diverse intermediates in synthesis, as they can be easily prepared from a variety of other functional groups [9]. Nucleophilic ring opening reactions, mediated by using suitable homogeneous acid catalysts [10], are of great importance in the preparation of polyfunctional compounds (mono-esters of diols) [11]. Intermediate ester compounds have a variety of industrial applications such as solvents, plasticisers, resins, plastics, coatings, perfumes, flavours, soaps, medicines, biofuels, and biolubricants [12]. The chemical modifications include acylation as a synthesis step to improve the biolubricant base stock properties. The acetyl compound reforms the carbonyl group with the loss of the chloride ion [13].

To make an improvement in the biolubricant properties such as low pour point and to increase biolubricant industrial stability, reactions by adding other esters group have been used [14]. In this present study, a novel synthetic approach has been chosen for modification of LA for improving the physical and tribological properties. Tribology is essential to modern edge apparatus, which surface interactions are extremely complex [15]. To understand the tribological interface requires comprehension of machine design, materials science and fluid mechanics. In general, the goal of this work is to indicate a new chemical biolubricant structure that might be synthesized by using LA as a base material which leads to suitable properties improvement, such as to pour point, flash point, viscosity and Tribological properties.

## Experimental and Methods

### Materials

All chemicals and solvents used in this study such as hexane, ethanol, hydrogen peroxide, toluene and acetic acid were from the analar grade and were used without further purification. Oleic acid (OA), Linoleic acid (LA), *p*-toluenesulfonic acid (PTSA), oleyl alcohol (OL) and oleoyl chloride (OLC) were acquired from Fisher and Merck.

### Synthesis Reactions

#### Monoepoxidation of linoleic acid (MELA) (2)

Linoleic acid **1** (1.4 g) was fluxed in 10 mL toluene and 120 mg of Novozym 435^®^ lipase was added to the solution. During stirring the reaction for 15 min, 30% H_2_O_2_ (15 μL) was added and the addition was repeated every 15 min for 7 h. after completed the reaction, the lipase Novozym 435^®^ was filtered and the mixture of reaction was washed with water and was dried by using the anhydrous sodium [16].

#### Synthesis of 9,(12)-hydroxy-10,(13)-oleioxyoctadecanoic acid (HYOODA) (3)

Compound **2** (1.55 g; 0.005 mol) and the catalyst PTSA (0.62 gm; 0.002 mol) were dissolved in toluene (10 mL) for 1.5 h and the temperature of the mixture reaction was adjusted at 50°C. OA (0.31 gm; 0.001 mol) was added during 1.5 h to keep the mixture temperature under 70–80°C. The reaction mixture was subsequently heated at 110°C, and for 4.5 h. The mixture was washed with the water and was dried by using anhydrous sodium sulphate [17].

#### Synthesis of oleyl 9,(12)-hydroxy-10,(13)-oleioxyoctadecanoate (OLHYOODT) (4)

Compound **3** (5 g; 0.01 mol) and OL of (10 g; 0.02 mol) were heated at 90°C for 1 h and then, the SG was adding to the reaction mixture. The mixture was heated at 110°C and for 7 h. The mixture was washed with the water and was dried by using anhydrous sodium sulphate [18].

#### Synthesis of oleyl 9,(12)-oleoyloxy-10,(13)-oleioxyoctadecanoate (OLOLOODT) (5)

Compound **4** (2.5g; 0.003 mol), pyridine (1.66 g; 0.002 mol) and CCl_4_ (10 mL) were mixed and heated at 60°C. OLC (16.2 g; 0.013 mol) was added during 1 h, and the reaction mixture was refluxed for (5.5 h). The mixture was washed with the water and was dried by using anhydrous sodium sulphate [19].

### Characterization

#### FTIR and ^1^H and ^13^C NMR

FTIR of the products was recorded on a Perkin Elmer Spectrum GX spectrophotometer in the range of 400–4000 cm^-1^. FTIR was used to measure functional groups of the synthesis products. A very thin film of products was applied to NaCl cells (25 mmi.d × 4 mm thickness) for analysis. ^1^H and ^13^C NMR analysis was performed with NMR spectroscopy model Joel FCP 400 MHz with the solvent CDCl_3_. ^1^H and ^13^C NMR of the products were recorded on a Bruker 300 NMR spectrophotometer [18].

#### Liquid Chromatography-Mass Spectroscopy Analysis

All the synthesis products were analyzed on hybrid QS-TAR Pulsar quadrupole TOF spectrometer equipped with an electrospray ionization source. The mobile phase solvents were methanol: acetic acid and water: acetic acid. An MS spectrum was recorded in the mass range of m/z 50–4000 amu and Nitrogen was used as curtain gas [20].

#### Pour point Cloud point and Flash point

PP value was measured according to the ASTM D5949 method [21] while the CP value was measured using the ASTM D5773 method [22], with a phase Technology Analyser, Model PSA-70S. FP value was run according to the American National Standard Method using a Tag Closed Tester (ASTM D 56–79) method [19].

#### Viscosity index

Multi-ranges viscometers obtained from Walter Herzog were used to measure the VI. The measurements have been done in a Temp-Trol (Precision Scientific). Viscometer bath set at 40.0 and 100.0°C and viscosity index (VI) was calculated using the ASTM methods [19].

#### Oxidative stability

Pressurized differential scanning calorimetry (PDSC) experiment was carried out using a DSC 2910 thermal analyser from TA Instruments (Newcastle, DE). PDSC was pressurized in the module at pressure of 1378.95 kPa (200 psi). A 10°C min^-1^ heating rate from 50 to 350°C was used during experiments. The oxidation onset (OT,°C) was calculated from a plot of the heat flow (W/g) versus temperature for each experiment [19].

#### Newtonian and Tribological Tests

Newtonian and tribological properties were performed according to ASTM D4172-94 method [23]. The tribological determinations were performed using the one-ball method (PCS Instruments, London, UK) via Laser Scientific (Granger, IN, USA) [19].

## Results and Discussion

### Synthesis of esters

In this study, triester derivative **5** was synthesized through a two-step reaction of monoepoxidation and opening of oxirane ring to synthesise the monoester 9,(12)-hydroxy-10,(13)-oleioxyoctadecanoic acid **3**. Product **2** results in a mixture of two monoepoxides (cis-9, 10-epoxy 12c- 18:1 (**2a**) and cis-12, 13 epoxy 9c- 18:1(**2b**)) with yield% of 82.14, while the oxirane ring opening in the presence of *p*-toluene sulfonic acid (PTSA) to prepare 9,(12)-hydroxy-10,(13)-oleioxyoctadecanoic acid **3** with yield% of 84.60 (Table 1).

**Table 1:** Variables and responses of synthesis compounds.

Variables	Compounds			
	2	3	4	5
H_2_O_2_ (μL)	15	-	-	-
Oleic acid (mol)	-	0.001	-	-
Oleyl alcohol (mol)	-	-	0.02	-
Oleoyl chloride (mol)	-	-	-	0.013
Novozyme (mg)	120	-	-	-
PTSA (mol)	-	0.002	-	-
Sulfric acid (mol)	-	-	0.007	-
Pyridine (mol)	-	-	-	0.002
Temperature (°C)	-	110	110	60
Time (h)	7	4.5	7	5.5
Responses	Compounds			
	**2**	**3**	**4**	**5**
Yield (%)	82.14	84.6	88.7	81.6
Conversation (%)	81.35	83.54	86.29	80.73
OOC (%)	4.91	0.05	-	-
IV (mg/g)	77.65	134.8	-	-

(**2**) Monoepoxidation of Linoleic Acid (MELA).

(**3**) 9,(12)-hydroxy-10,(13)-oleioxyoctadecanoic acid (HYOODA).

(**4**) Oleyl 9,(12)-hydroxy-10,(13)-oleioxyoctadecanoate (OLHYOODT).

(**5**) Oleyl 9,(12)-oleoyloxy-10,(13)-oleioxyoctadecanoate (OLOLOODT).

**Fig 1:**
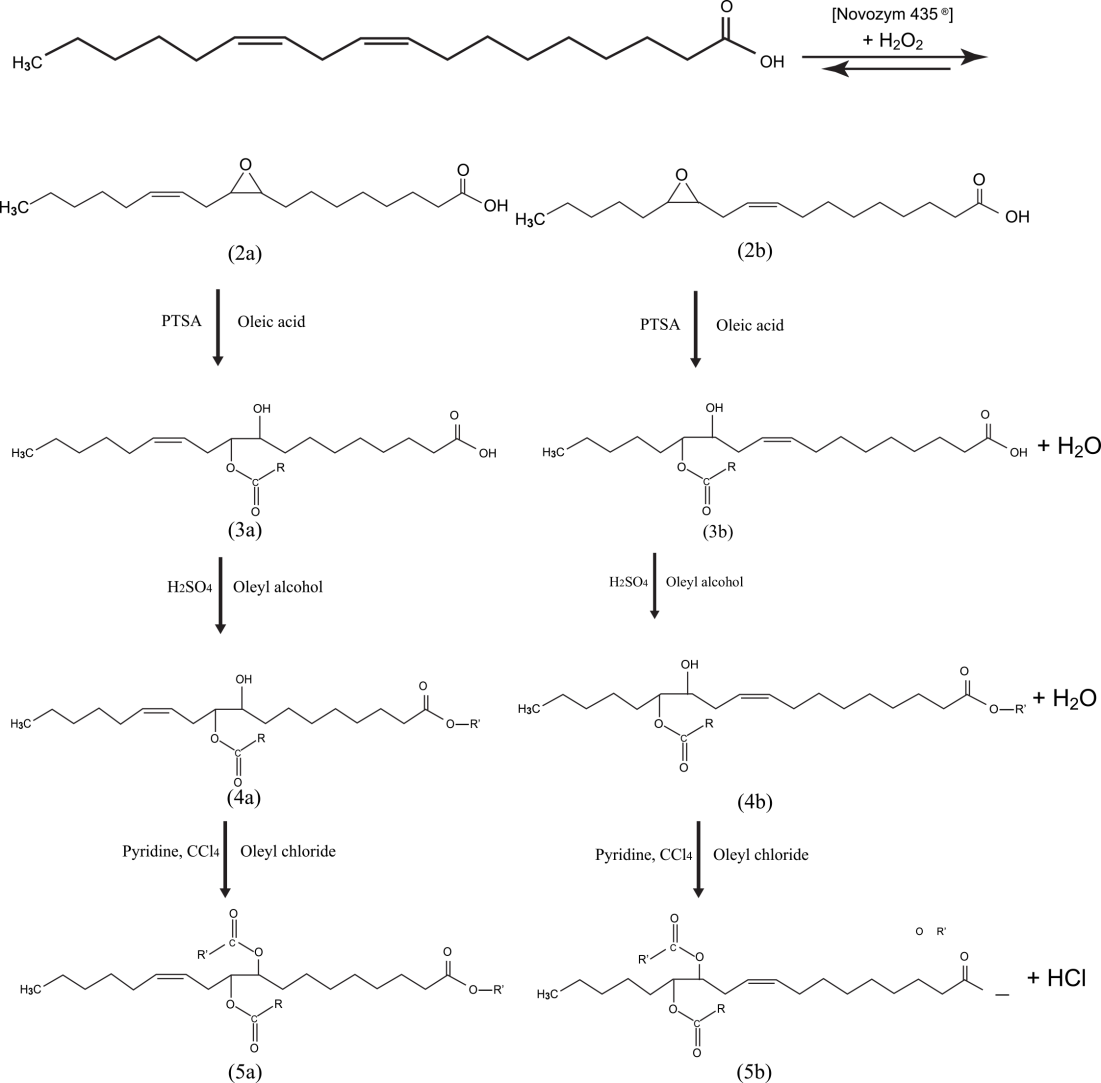
Reaction scheme for the formation of triester. (2a, 2b) cis-9, 10-epoxy 12c- 18:1 and cis-12, 13 epoxy 9c- 18:1. (3a, 3b) 9,(12)-hydroxy-10,(13)-oleioxyoctadecanoic acid. (4a, 4b) oleyl 9,(12)-hydroxy-10,(13)-oleioxyoctadecanoate. (5a, 5b) oleyl 9,(12)-oleoyloxy-10,(13)-oleioxyoctadecanoate.

In addition, the second two-step reaction has been done using esterification and acetlytion reactions. Oleyl 9,(12)-hydroxy-10,(13)-oleioxyoctadecanoate **4** was synthesized by using carboxylic acid with oleyl alcohol with yield% of 88.77 (Table 1). Finally, acetlytion of α-hydroxy group in diester **4** with oleoyl chloride yielded 81.60% (Table 1) of triester oleyl 9,(12)-oleoyloxy-10,(13)-oleioxyoctadecanoate **5** (Fig 1).

Based on earlier researches, the findings demonstrated that by expanding the mid-chain of ester group length enhanced the low pour point; however had a negative impact on the oxidative stability [16]. The results showed low pour point of compound **2** at -51°C. Moreover, the low pour point of plant oil prevents its use at low operating temperatures, however by introducing branch sites at the epoxy carbons is a good strategy to enhance the pour point property. The branched products have importantly enhanced tribological properties such as friction-wear compared with the compound **2** [18].

### Characterization

#### FTIR

The spectrum from the FTIR analysis displays several absorption peaks. The main peaks and their assignment to functional groups are given in Table 2. The FTIR spectrum of compound **2** shows the peak of an epoxy group at 820 cm^−1^. The other important peaks observed in the FTIR spectrum are: 720 cm^−1^ (methylene) and 1711 cm^−1^ (C = O stretch). In the FTIR spectra of synthesis compounds (**3**–**5)**, the absorption bands from the epoxy group (820 cm^−1^) were not observed. This result suggests that with compound **2**, there is a complete ring opening under the reaction conditions. Functional groups representing C = O (1737, 1738 cm^−1^), CH_3_ (1373–1460 cm^−1^), OH groups (3413–3445 cm^−1^) and the C-O bands of esters (1117–1118 cm^−1^) are clearly visible in the spectra.

**Table 2:** The main FTIR functional groups wavenumbers of linoleic acid and synthesis compounds.

Functional groups	Compounds				
	1	2	3	4	5
C = O stretching vibration (carboxylic acid)	1719	1711	1711	-	-
C-O-C oxirane ring	-	820	-	-	-
OH stretching (alcohol)	-	-	3413	3445	-
C = O stretching vibration (ester)	-	-	1737	1738	1738
C-O bending vibration (ester)	-	-	1117	1117	1118

(**1**) Linoleic acid (LA).

(**2**) Monoepoxidation of Linoleic Acid (MELA).

(**3**) 9,(12)-hydroxy-10,(13)-oleioxyoctadecanoic acid (HYOODA).

(**4**) Oleyl 9,(12)-hydroxy-10,(13)-oleioxyoctadecanoate (OLHYOODT).

(**5**) Oleyl 9,(12)-oleoyloxy-10,(13)-oleioxyoctadecanoate (OLOLOODT).

#### ^1^H and ^13^C NMR

**Fig 2:**
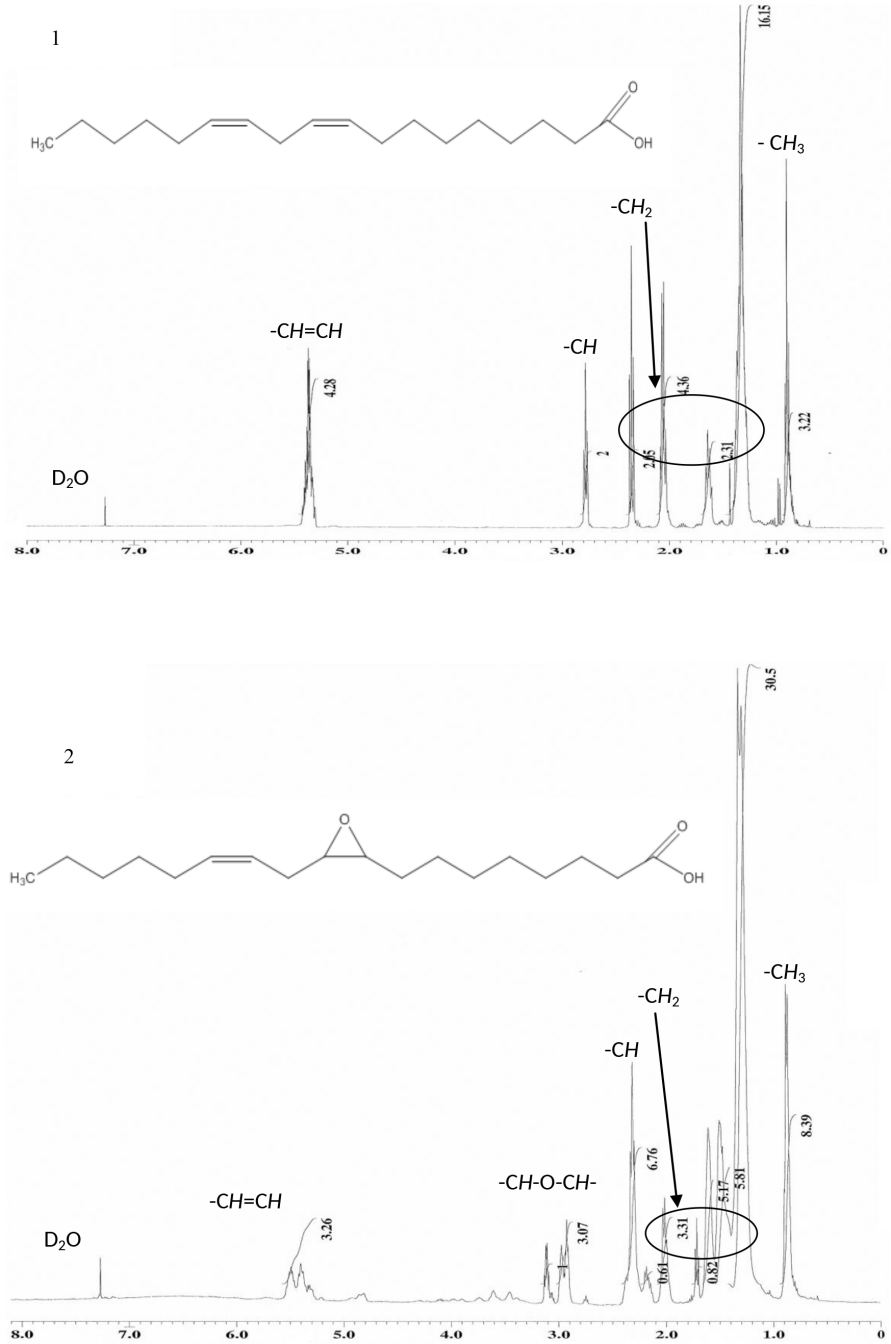
^1^H NMR spectrums of linoleic acid and synthesis compounds. (1) Linoelic acid. (2) Monoepoxidation. (3) Oxirane ring opening. (4) Esterification. (5) Acetlyation.

The structures of all synthesized compounds **1**, **2**, **3**, **4** and **5** were confirmed by ^1^H and ^13^C NMR spectroscopy. Significant signals in the ^1^H spectrum of compound **2** at 2.92–3.12 ppm were due to the tertiary hydrogens of the oxirane ring; while the main signal in HYOODA **3** was 3.62 and 4.06 ppm for hydrogens of the alcohol and ester, respectively. Furthermore, the significant signals of compound **4** and OLOLOODT **5** were 4.05 ppm for hydrogens of the esters (Fig 2).

**Fig 3:**
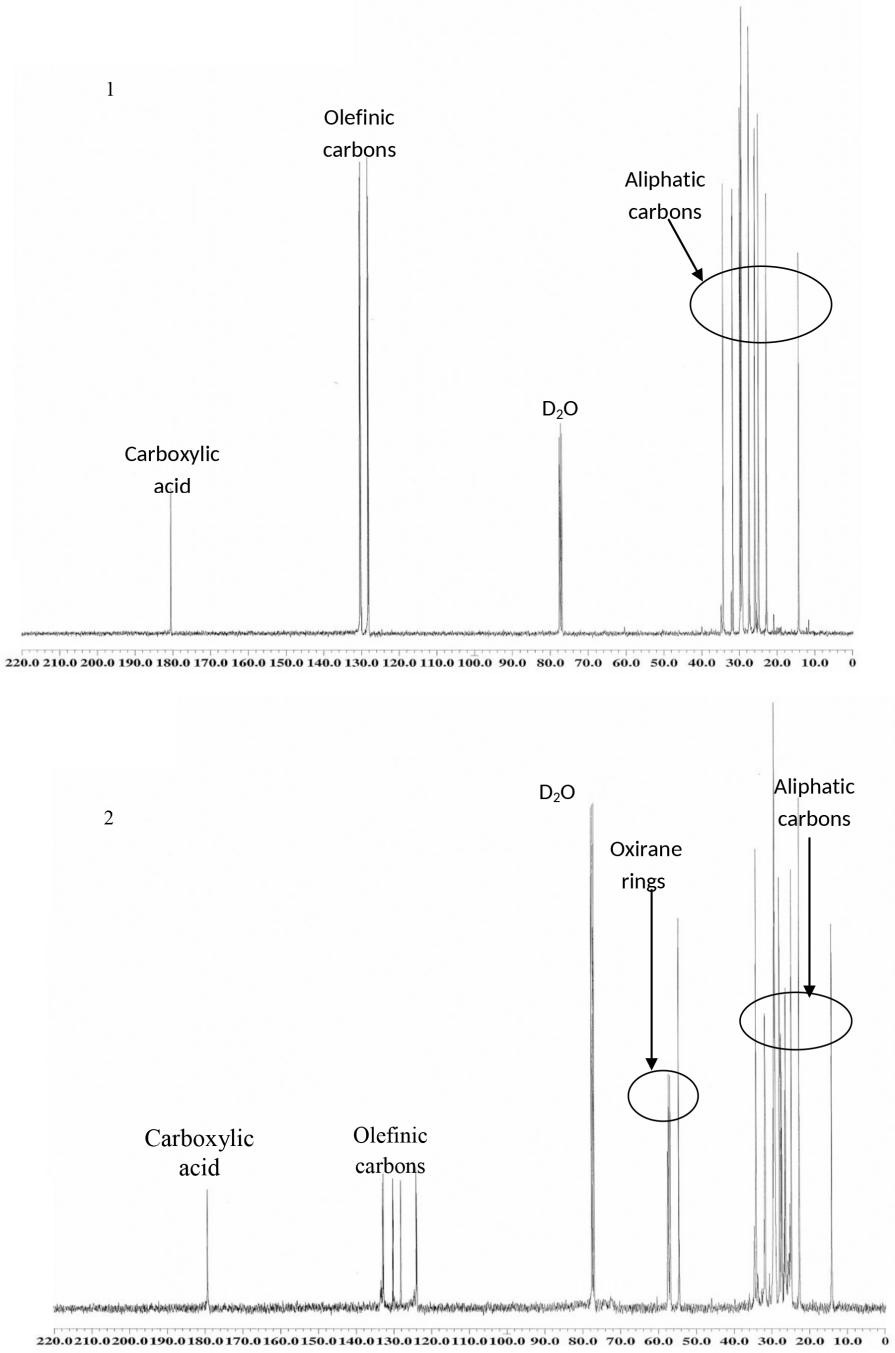
^13^C NMR spectrums of linoleic acid and synthesis compounds. (1) Linoelic acid. (2) Monoepoxidation. (3) Oxirane ring opening. (4) Esterification. (5) Acetlyation.

In the ^13^C spectrum of compound **2**, the signals at 54.69–57.29 ppm were due to the carbons of the oxirane ring; while the main signal in compound **3** was 64.41 ppm for the alcohol carbon. Furthermore, in the ^13^C NMR spectra, the signals of compounds **4** and **5** were 173.93 and 173.89 ppm which attributed to the ester carbonyl groups, respectively (Fig 3) [24].

#### LC-MS

Synthetic products upon fractional **1**, **2**, **3**, **4** and **5** were approved by liquid chromatography mass spectroscopy detection (LC-MS) Table 3. The LC-MS provide evidence for the proposed structures. The negative mode was operated by using ion source to generate [*M–H*]^-^, which are procumbent to charge-distant shatter [20]. Charge-distant shatter of fatty acids occurs to give both an alkenes and charged species possessing the carboxylic group [25].

**Table 3:** LC-MS for the synthetic compounds and data used for their identification.

Compounds	Molecular [*M–H*]^-^	No. of double bonds
**1**	280.2335	2
**2**	296.2278	1
**3**	578.4627	2
**4**	829.3917	3
**5**	1093.8403	4

(**1**) Linoleic acid (LA).

(**2**) Monoepoxidation of Linoleic Acid (MELA).

(**3**) 9,(12)-hydroxy-10,(13)-oleioxyoctadecanoic acid (HYOODA).

(**4**) Oleyl 9,(12)-hydroxy-10,(13)-oleioxyoctadecanoate (OLHYOODT).

(**5**) Oleyl 9,(12)-oleoyloxy-10,(13)-oleioxyoctadecanoate (OLOLOODT).

**Fig 4:**
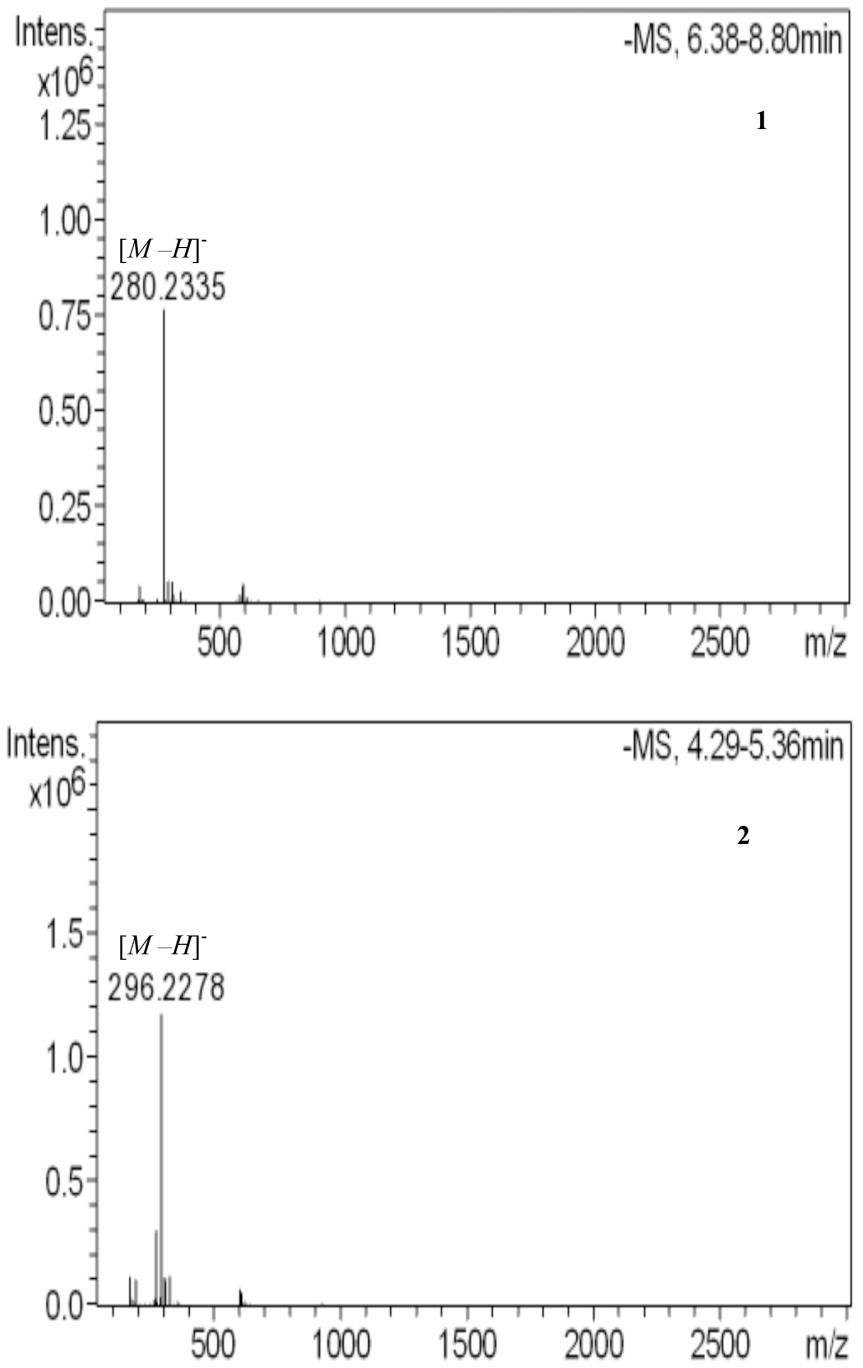
LC-MS analysis of linoleic acid and synthesis compounds. (1) Linoelic acid. (2) Monoepoxidation. (3) Oxirane ring opening. (4) Esterification. (5) Acetlyation.

Table 3 demonstrates the molecular weight [*M—H*]^-^ for the synthetic products, while the LC-MS spectrums are shown in Fig 4. LC-MS spectra obtained upon charge-distant shatter is typically clear to interpret and have been widely used for organic synthetic structural determination of fatty acids and others related ester compounds [26].

### Physicochemical properties

Physicochemical properties include low temperature behavior (cloud point (CP), pour point (PP)), flash point (FP), viscosity index (VI) and oxidative stability (OT) (Table 4). It decades the capability of plant oil as a biolubricant, based on the processing technology. CP and PP show the suitability of biolubricant in cold weather conditions. Biolubricant used in industrial machines working at low temperatures should have low pour point; otherwise waxes of biolubricants will cause in the machines because of waxes in the biolubricant increase pour point. A poor PP limits the variety of industrial application of plant oils at low temperatures [27, 28]. Compound **2** has a PP of 15°C and a CP of 13°C. All the synthesized esters (**3**–**5**) have better PPs, in the range of -51 to -73°C, and CPs in the range of -57 to -70°C (Table 4). Attachment of OLC to synthesis triester (**5**) was effective in decreasing the PP and CP to -73 and -70°C, respectively. The results showed that the high branching sites on the LA forms a steric barrier around the molecules and inhibits solidifications, which resulted in lower PP [29].

**Table 4:** Physicochemical characteristics of synthesis compounds.

Compounds	Density (g/cm3)	Volatility 120°C (%)	Pour point (°C)	Cloud point (°C)	Flash point (°C)	Viscosity index	Oxidative stability (°C)
**2**	0.872	0.50	15	13	128	130	168
**3**	0.876	0.43	-51	-57	251	153	180
**4**	0.879	0.35	-62	-69	264	192	215
**5**	0.882	0.30	-73	-70	279	219	260

(**2**) Monoepoxidation of Linoleic Acid (MELA).

(**3**) 9,(12)-hydroxy-10,(13)-oleioxyoctadecanoic acid (HYOODA).

(**4**) Oleyl 9,(12)-hydroxy-10,(13)-oleioxyoctadecanoate (OLHYOODT).

(**5**) Oleyl 9,(12)-oleoyloxy-10,(13)-oleioxyoctadecanoate (OLOLOODT).

FP is a gainful way to determine the volatility, transportation, fire resistance and storage temperature requirements for biolubricants base stocks. The FP should be high for safety operation and minimal volatilization at the maximum temperature. In addition, some demanding industrial applications, such as an aviation jet engine, the effective biolubricant base stocks range of over 300°C may be required [16]. Table 4 shows the improvement in FP of triester (**5**), which increases to 279°C, which that agrees with the other international standards. Overall, the FP increases with the increasing of the molecular weight.

VI describes how oil reacts to viscosity temperature changes for 40°C and 100°C. Motor biolubricant that displays an extensive viscosity change between two temperatures has a low VI, while other types of biolubricants have a higher VI. The outcomes demonstrated that the VI increases as the exchange of the oil increased in the order monoester (**3**) < diester (**4**) < triester (**5**) (Table 4). Furthermore, biolubricant with higher VI shows low viscosity changes with temperatures and is considered to have a stable viscosity- temperature relation [30].

The results showed by using OLC for acylation of diester (**4**), significantly improves the oxidation stability of OT for triester (**5**) at 260°C more than OLHYOODT 215°C (Table 4). These synthesis ester compounds, monoester (**3**) < diester (**4**) < triester (**5**) are in agreement with other studies on synthetic esters, in which the oxidative stability increases with the increasing chain length of the esterified FA [31]. A high OT suggests a high oxidation stability of triester (**5**).

### Tribological properties

**Fig 5:**
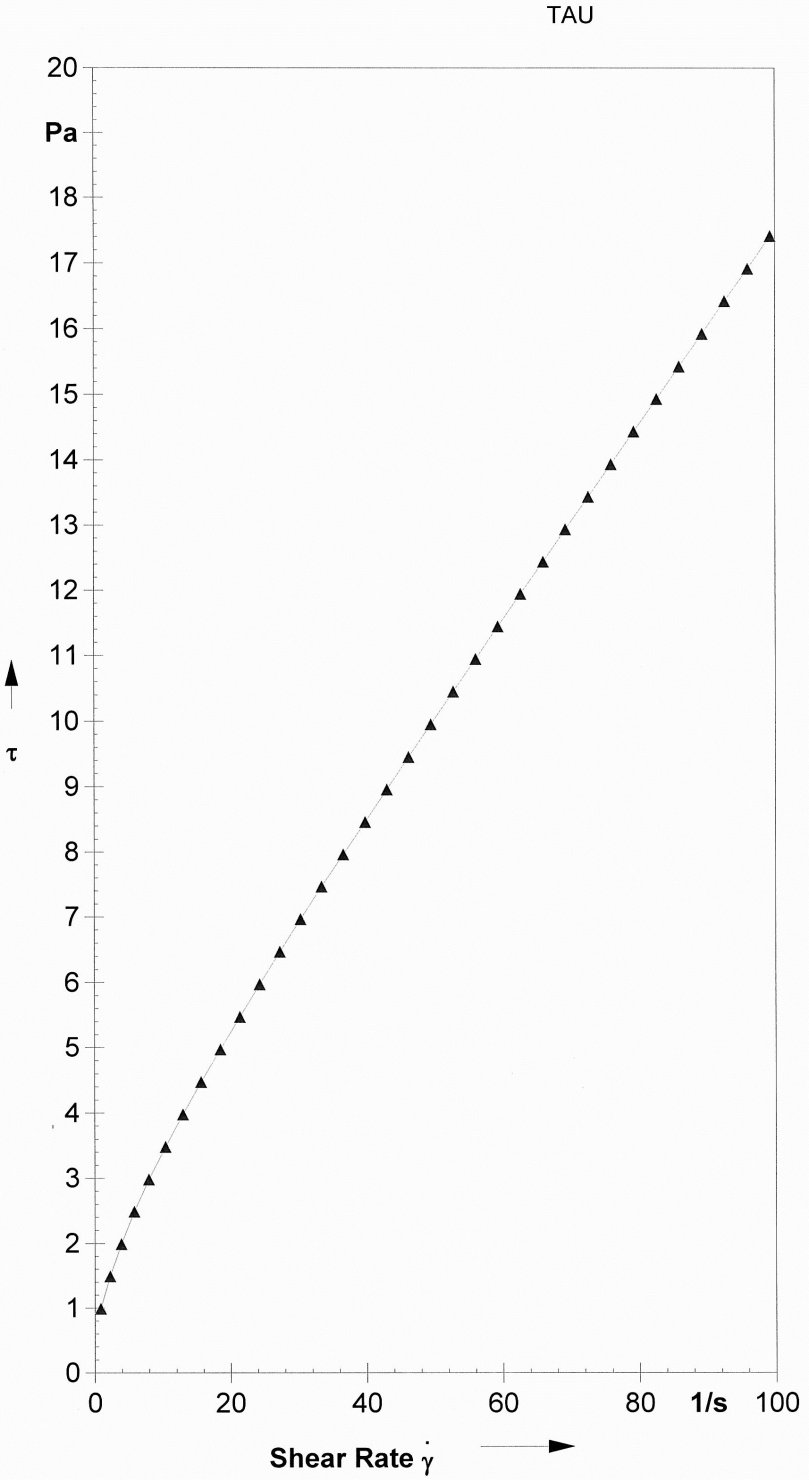
Newtonian rheological of triester. The relation between the shear stress (τ) and shear. rate (γ).

Dynamic viscosity is essential to evaluate the viability of viscosity. Fig 5 demonstrates the connection between the shear stress (τ) and shear rate (γ) which is straight as the shear rate increases [32]. In a Newtonian liquid, if the rate, load and temperature increase, which lead to the increase of the rate of diffusion of oil molecules, interaction with surfaces and number of active sites; moreover, Newtonian liquids are utilized to increase the biolubricants VI by including new branches in synthesized compounds [33].

**Fig 6:**
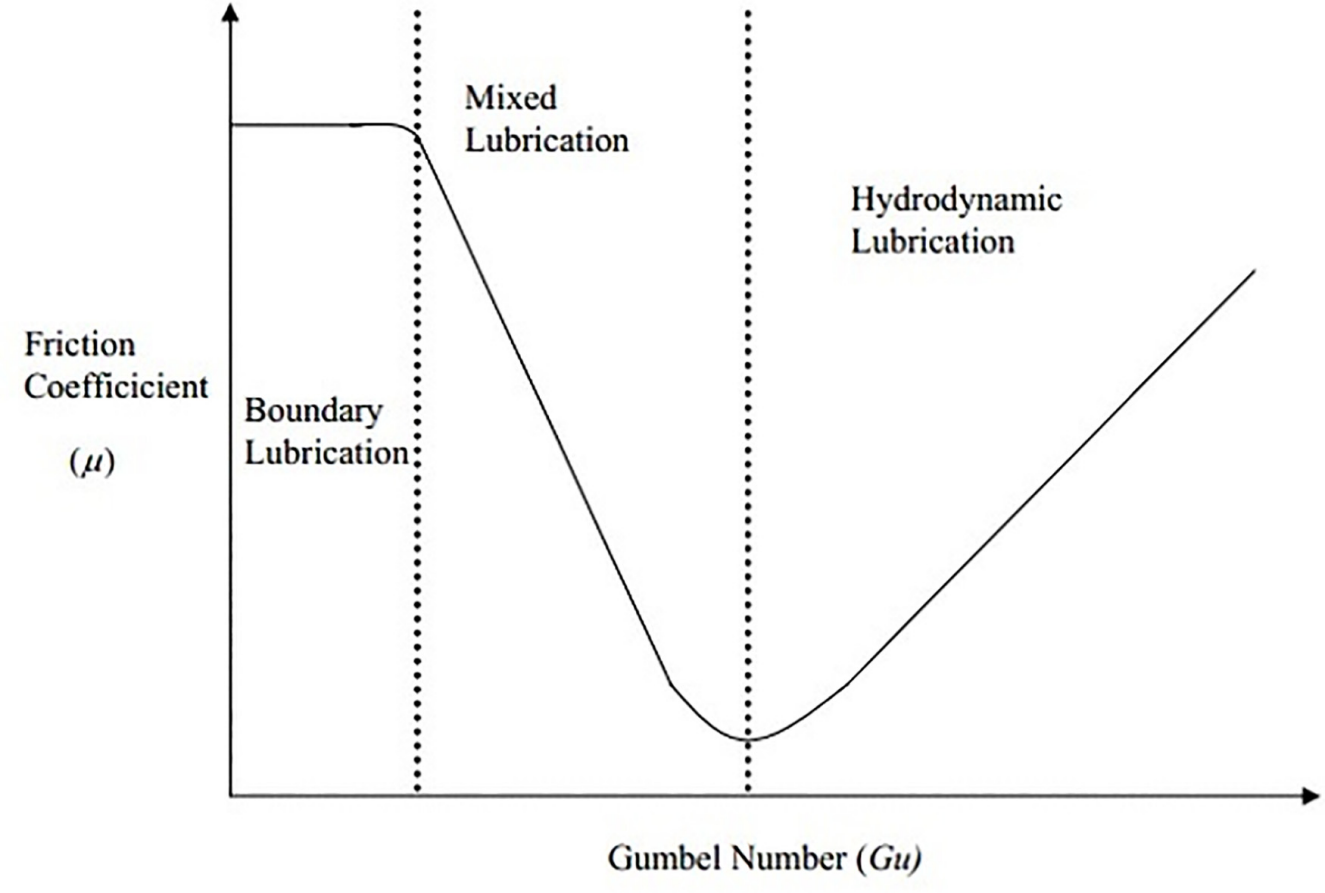
Stribeck curve. Dynamic viscosity ŋ, shaft rotation rate ω, and mean pressure *P* and the friction coefficient μ for a typical rotating bearing. Lubrication modes of a system are related to asperity-asperity interaction and fluid separation of two surfaces as suggested by the inset cartoons.

Synthesized new structural compounds for biolubricant oil can be classified in several different modes such as boundary, mixed and hydrodynamics. Fig 6 demonstrates the stribeck curve which mentioned the relationships between different modes [34]. Boundary mode shows the mechanism of lubrication which provided by thin layers formed on contacting surface austerity by chemical or physical adsorption [35], while for surface chemistry and fluid machines which contribute to friction has been shown in mixed mode shares characteristics [34]. Furthermore, the surfaces which were separated by a thick film of fluid and friction are shown in hydrodynamic mode [35].

The results demonstrated that the triester compound (**5**) can be used as both boundary and hydrodynamic biolubricants [36]. The newtonian presumption that the shear stress in a fluid is comparative to the shear rate molder under the pressures. The results demonstrate high viscosity for the synthesized structural compounds which lead to the shear stress to exceed the stress of steel [37]. Moreover, increasing the viscosity causes dropping in coefficient of friction and increasing the speed makes the rate of transition to biolubricant increases [23]. The friction coefficients measurements versus sliding speed are given in (Table 5). In general, the results demonstrated that by increasing the pressure causes increasing in viscosity while increasing in temperatures will makes fall in viscosity (Table 4).

**Table 5:** Tribological characteristics of synthesis compounds.

Compounds	Coefficient of friction (μ)			
	Maximum 40°C	Maximum 100°C	Minimum 40°C	Minimum 100°C
**2**	0.95	0.79	0.17	0.12
**3**	0.54	0.65	0.13	0.18
**4**	0.46	0.62	0.10	0.14
**5**	0.44	0.57	0.15	0.20

(**2**) Monoepoxidation of Linoleic Acid (MELA).

(**3**) 9,(12)-hydroxy-10,(13)-oleioxyoctadecanoic acid (HYOODA).

(**4**) Oleyl 9,(12)-hydroxy-10,(13)-oleioxyoctadecanoate (OLHYOODT).

(**5**) Oleyl 9,(12)-oleoyloxy-10,(13)-oleioxyoctadecanoate (OLOLOODT).

### Conclusion

This study describes the impact of an ester branches on the properties of biolubricants oil. This goal has been achieved by increasing the molecular structure of triester product (**5**). Increasing the chain length of the ester branches and the hydrogen bonding had a positive effect on the viscosity index, oxidative stability, pour point, newtonian and tribological properties of the new synthesis compounds. Furthermore, increasing the molecular structure of the biolubricant had resulted in a positive effect on wear protection.

## References

[1] Rani S, Joy ML, Nair KP. Evaluation of physiochemical and tribological properties of rice branoil biodegradable and potential base stoke for industrial lubricants. Industrial Crops and Products. 2014; 65: 328–333. 10.1016/j.indcrop.2014.12.020

[2] Asadauskas S, Perez JM, Duda JL. Oxidative stability and antiwear properties of high oleic vegetable oils. Lubrication Engineering. 1996; 52: 877–882.

[3] Erhan SZ, Asadauskas S. Lubricant basestocks from vegetable oils. Industrial Crops and Products. 2000; 11: 277–282. 10.1016/S0926-6690(99)00061-8

[4] Özgülsün A, Karasmanoğlu F; Tüter M. Esterification reaction of oleic acid with a fusel oil fraction for production of lubricating oil. Journal of the American Oil Chemists’ Society. 2000; 77: 105–109. 10.1007/s11746-000-0017-5

[5] Syaima MTS, Ong KH, Noor IM, Zamratul MIM, Brahim SA, Hafizul MM. The synthesis of biolubricant based oil by hydrolysis and non-catalytic of palm oil mill effluent (POME) using lipase. Renewable and Sustainable Energy Reviews. 2015; 44: 669–675. 10.1016/j.rser.2015.01.005

[6] Hwang H-S, Erhan SZ. Synthetic lubricant basestocks from epoxidezed soybean oil and Guerbet alcohols. Industrial Crops and Products. 2006; 23: 311–317. 10.1016/j.indcrop.2005.09.002

[7] Janković MR, Snežana V, Fišer S. Kinetic models of reaction systems for the in situ epoxidation of unsaturated fatty acid esters and triglycerides. Chem. Ind. 2004; 58: 569–576. 10.2298/HEMIND0412569J

[8] Salimon J, Abdullah BM, Yusop RM, Salih N. Synthesis, reactivity and application studies for different biolubricants. Chemistry Central journal. 2014; 8:16 10.1186/1752-153X-8-16 24612780 PMC3995787

[9] Ready JM, Jacobsen EN. A practical oligomeric [(salen)Co] catalyst for asymmetric epoxide ring-opening reactions. Angew. Chem. Int. Ed. Engl. 2002; 41, 1374–1377. 10.1002/1521-3757(20020415)114:8<1432::AID-ANGE1432>3.0.CO;2-6 19750769

[10] Yunus R, Fakhru’l-Razi A, Ooi TL, Iyuke SE, Idris A. Development of optimum synthesis method for transesterification of palm oil methyl esters and trimethylolpropane to environmentally acceptable palm oil-based lubricant. Journal of Oil Palm Research. 2003; 15: 35–41.

[11] Méndez PS, Cachau RE, Seoane G, Ventura ON. Regioselective epoxide ring-opening using boron trifluoride diethyl etherate: DFT study of an alternative mechanism to explain the formation of syn-fluorohydrins. Journal of Molecular Structure: THEOCHEM. 2009; 904: 21–27. 10.1016/j.theochem.2009.02.023

[12] Kaufmann AJ, Ruebusch RJ. Oleochemicals-a look at world trends. INFORM. 1990; 1:1034–1048.

[13] Cary FA, Sundberg RJ. Advanced organic chemistry part A: Structure and mechanisms, (Fifth Ed.). Springer, University of Virginia, USA, 2007.

[14] Sharma BK, Adhvaryu A, Liu Z, Erhan SZ. Chemical modification of vegetable oils for lubricants applications. Journal of the American Oil Chemists’ Society. 2006; 83: 129–136. 10.1007/s11746-006-1185-z

[15] Patil VR, Jadhav MM, Pawar GB, Gunjavate PV. Some studies on tribological properties of lubricating oil with nanoparticles as an additive. International Journal of Advanced Engineering Technology. 2014; 01–04.

[16] Salimon J, Salih N, Abdullah BM. Production of chemoenzymatic catalyzed monoepoxide biolubricant: optimization and physicochemical characteristics. Journal of Biomedicine and Biotechnology. 2012; Volume 2012, Article ID 693848. 10.1155/2012/693848 PMC3278930 22346338

[17] Salimon J, Abdullah BM, Yusop RM, Salih N. Synthesis and optimization ring opening of monoepoxide linoleic acid using p-Toluenesulfonic acid. SpringerPlus. 2013; 2013, 2:429 10.1186/2193-1801-2-429 24083099 PMC3786081

[18] Salimon J, Abdullah BM, Salih N. Diesters biolubricant base oils: synthesis, optimization, characterization and physicochemical characteristics. International Journal of Chemical Engineering. 2012; Volume 2012, Article ID 896598. 10.1155/2012/896598

[19] Salih N, Salimon J, Yousif E, Abdullah BM. Biolubricant basestocks from chemically modified plant oils: ricinoleic acid based-tetraesters. Chemistry Central Journal. 2013; 7: 128 10.1186/1752-153X-7-128 23885790 PMC3726387

[20] Orellana-Coca C, Adlercreutz D, Andersson MM, Mattiasson B, Hatti-Kaul R. Analysis of fatty acid epoxidation by high performance liquid chromatography coupled with evaporative light scattering detection and mass spectrometry. Chemistry and Physics of Lipids. 2005; 135: 189–199. . 15921978 10.1016/j.chemphyslip.2005.02.014

[21] American Society for Testing Materials. Standard Test Method for Pour Point of Petroleum (Automatic Pressure Pulsing Method). ASTM, West Conshohocken, PA, USA, 2011a.

[22] American Society for Testing Materials. Standard Test Method for Flash Point of Liquids with a Viscosity Less Than, 45 Saybolt Universal Seconds (SUS) at 37.8°C (that don’t contain suspended solids and don’t tend to form a surface film under test), 2011b.

[23] Kosanov J, Lenard JG, Uhrig J, Wallfarth B. The effect of lubricant additives on the coefficient of friction in the flat-die test. Materials Science and Engineering A. 2006; 427: 274–281. 10.1016/j.msea.2006.04.090.

[24] Sliverstien R, Bassler G, Morrill T. Spectrometric Identification of Organic Compounds, 7th ed. John-Wiley, New York, 2005.

[25] Cheng C, Pittenauer E, Gross M. Charge-remote fragmentations are energy-dependent processes. Journal of the American Society for Mass Spectrometry. 1998; 9: 840–844. . 9692256 10.1016/S1044-0305(98)00053-1

[26] Hsu F-F, Turk J. Studies on sulfatides by quadrupole ion-trap mass spectrometry with electrospray ionization: structural characterization and the fragmentation processes that include an unusual internal galactose residue loss and the classical charge-remote fragmentation. Journal of the American Society for Mass Spectrometry. 2004; 15:536–546. 10.1016/j.jasms.2003.12.007 15047058

[27] Yao L, Hammond EG, Wang T, Bhuyan S, Sundararajan S. Synthesis and physical properties of potential biolubricants based on ricinoleic acid. Journal of the American Chemical Society. 2010; 87, 937–945. 10.1007/s11746-010-1574-1

[28] Hwang H, Erhan SZ. Modification of epoxidized soybean oil for lubricant formulations with improved oxidative stability and low pour point. Journal of the American Oil Chemists’ Society. 2001; 78: 1179–1184. 10.1007/s11745-001-0410-0

[29] Sharma BK, Doll KM, Erhan SZ. Ester hydroxy derivatives of methyl oleate: tribological, oxidation and low temperature properties. Bioresource Technology. 2008; 99: 7333–7340. 10.1016/j.biortech.2007.12.057 18242085

[30] Hwang HS, Adhvaryu A, Erhan SZ. Preparation and properties of lubricant basestocks from epoxidized soybean oil and 2-ethylhexanol. Journal of the American Oil Chemists’ Society. 2003; 80: 811–815. 10.1007/s11746-003-0777-y

[31] Kubouchi H, Kai H, Miyashita K, Matsuda K. Effects of emulsifiers on the oxidative stability of soybean oil TAG in emulsions. Journal of the American Oil Chemists’ Society. 2002; 79: 567–570. 10.1007/s11746-002-0523-5

[32] Höglund E. Influence of lubricant properties on elastohydrodynamic lubrication. Wear. 1999; 232: 176–184. 10.1016/S0043-1648(99)00143-X

[33] Nessil A, Larbi S, Belhaneche H, Malki M. Journal Bearings Lubrication Aspect Analysis Using Non-Newtonian Fluids. Advances in Tribology. 2013; 212568, 9 10.1155/2013/212568

[34] Gleghorn JP, Bonassar LJ. Lubrication mode analysis of articular cartilage using stribeck surfaces. Journal of Biomechanics. 2008; 41: 1910–1918. 10.1016/j.jbiomech.2008.03.043 18502429

[35] Reyes EAA, Fisics L. Rheology and ferrohydrodynamic lubrication of magnetic fluids. Ph.D thesis, University of Granada, Department of applied physics, Granada, Spain, 2011.

[36] Castro W, Weller DE, Cheenkachorn K, Perez JM. The effect of chemical structure of base fluids on antiwear effectiveness of additives. Tribology International. 2005; 38:321–326. 10.1016/j.triboint.2004.08.020

[37] Greenwood JA, Kauzlarich JJ. Elastohydrodynamic film thickness for shear-thinning lubricants. J. Engineering Trib. 1998; 212: 179–191. 10.1243/1350650981541994

